# 371. Social Determinants of Vaccine Uptake: The Impact of Race and Vulnerability on Influenza, SARS-CoV-2, and RSV Vaccination Rates

**DOI:** 10.1093/ofid/ofae631.112

**Published:** 2025-01-29

**Authors:** Zainab Albar, Christopher Ladikos, Daniel J Tisch, Robert Salata, Elie Saade

**Affiliations:** Case Western Reserve University, Cleveland, Ohio; University Hospitals, Brunswick, Ohio; Case Western Reserve University, Cleveland, Ohio; Case Western Reserve University Hospitals, Cleveland, OH; Case Western Reserve University, Cleveland, Ohio

## Abstract

**Background:**

U.S. vaccination rates fall short, with social vulnerabilities hindering access to preventive care. The influence of social determinants on vaccine uptake is a critical public health concern. This study explored the connection between the social vulnerability index (SVI), race, and vaccination rates for Influenza, SARS-CoV2, and RSV.

**Figure 1:**
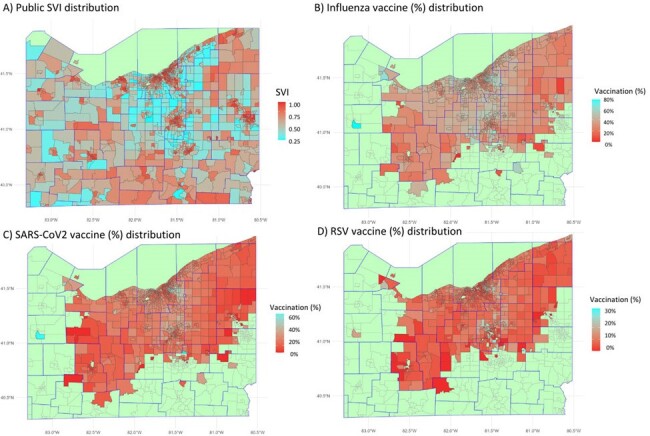
Geospatial Distribution of Social Vulnerability Index and Vaccination Rates for Influenza, SARS-CoV-2, and RSV across Ohio Census Tracts with Patients to the University Hospital Healthcare Network.

Geospatial representation of A) Public Social Vulnerability Index (SVI) for Ohio census tracts in 2020, B) Percentage of influenza vaccination, C) Percentage of SARS-CoV-2 vaccination, and D) Percentage of RSV vaccination for individuals aged 60 years and above. Figures B, C, and D show census tracts with a minimum of 10 patients presenting with mild to moderate respiratory infections to the University Hospital Healthcare network. The color scale for SVI ranges from 0 to 1, with the darkest red indicating the most vulnerable (SVI = 1) and the brightest cyan representing the least vulnerable (SVI = 0). The color scale for vaccination percentages spans from 0 to 100, with the darkest red denoting 0% vaccinated and the brightest cyan signifying 100% vaccinated.

**Methods:**

We analyzed data from patients with acute respiratory infections treated at University Hospitals of Cleveland from October 2023 to April 2024. Patient demographics and vaccination statuses were collected from health records and matched with SVI census tract-level public data, which was geocoded and segmented into quartiles. We employed the Kruskal-Wallis and Pearson's Chi-squared tests for statistical analysis, and multivariate logistic regression adjusted for age and sex to assess vaccination odds. Results were depicted using geospatial methods to show the distribution of vaccinations and SVI across Ohio.

**Figure 2:**
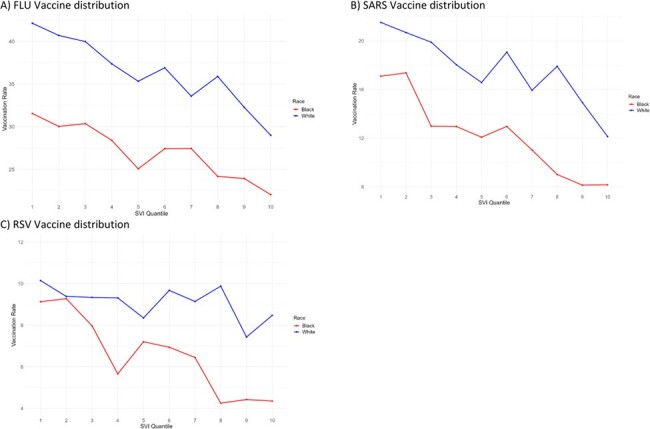
Vaccinations Rates across race and social vulnerability index quantiles

A comparison of vaccination rates for A) influenza, B) SARS-CoV-2, and C) RSV between White and Black populations across 10 quantiles of the Social Vulnerability Index (SVI). The SVI quantiles are represented on the x-axis, range from 0 to 1, with quantile 1 (SVI ≤ 0.2) representing the least vulnerable population and quantile 10 (SVI ≥ 0.8) representing the most vulnerable population. The y-axis shows the vaccination rates for each respective quantile. The color scheme used in the figure shows the White population in blue and the Black population in red. The summary statistics provide insights into the disparities in vaccination rates between White and Black individuals across varying levels of social vulnerability.

**Results:**

The study analyzed a diverse population of 341,029 individuals, with 60% female and 38% male participants, and primarily consisted of white individuals (81%). Black individuals made up 14% of the population and were disproportionately represented in the highest vulnerability quartile (Q4) at 37%, which was significantly higher than the 3.2% in the lowest quartile (Q1). As the SVI quartile increased, vaccination rates decreased, particularly among Black individuals. Additionally, emergency visits for ARIs increased, while primary care visits decreased with higher SVI quartiles. The likelihood of vaccination decreased as the SVI quartile increased, especially among the Black group. The odds of receiving a flu vaccine were lower for Black individuals compared to White individuals in all SVI quartiles, with the greatest disparity in SVI Q5. Similarly, the odds of receiving the SARS-CoV-2 vaccine and the RSV vaccine were also lower for Black individuals compared to White individuals in all SVI quartiles. Detailed results are presented in the associated tables and figures.

**Table 1:**
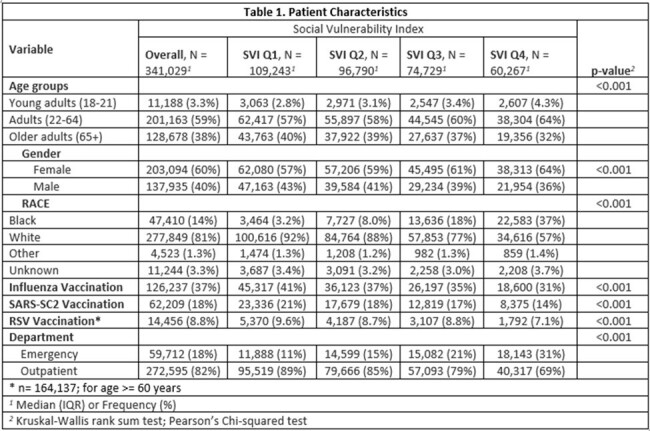
Study Population Characteristics Across Social Vulnerability Index Quartiles

**Conclusion:**

Socioeconomic vulnerability and race impact vaccination behaviors, emphasizing the need for tailored interventions to reduce disparities in vaccination rates among diverse populations.

**Table 2:**
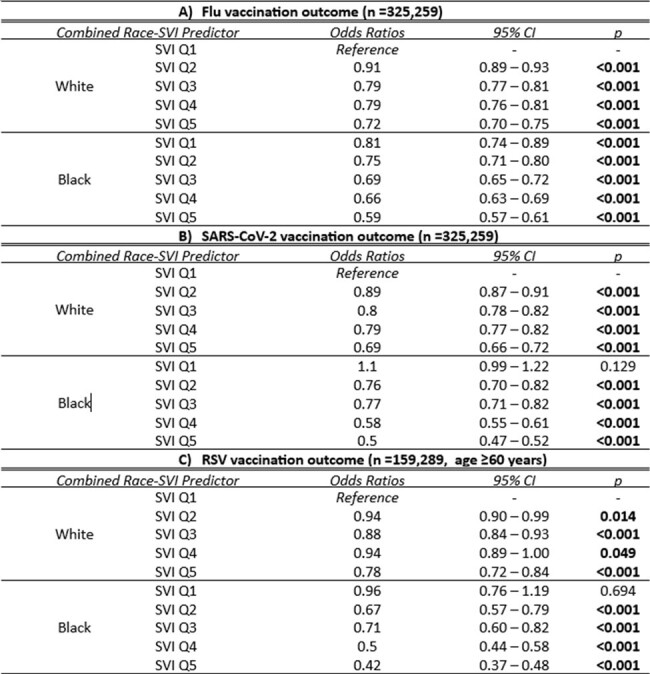
Multivariate associations between Social Vulnerability Index (SVI) and Vaccination within White and Black Race Population

Multivariate logistic regression models were used to evaluate the combined association between the SVI and race with the likelihood of vaccination for A) Influenza, B) SARS-CoV-2, and C) RSV. The models were adjusted for age and gender. The reference group for the combined race-SVI predictor was White individuals in the least vulnerable SVI quintile (Q1). Odds ratios, 95% confidence intervals, and p-values are presented for each race-SVI combination.

**Disclosures:**

**Elie Saade, MD, MPH, FIDSA**, Janssen Global Services: Advisor/Consultant|Janssen Research and Development: Advisor/Consultant

